# Information-Theoretic Approaches in EEG Correlates of Auditory Perceptual Awareness under Informational Masking

**DOI:** 10.3390/biology12070967

**Published:** 2023-07-06

**Authors:** Alexandre Veyrié, Arnaud Noreña, Jean-Christophe Sarrazin, Laurent Pezard

**Affiliations:** 1 Centre National de la Recherche Scientifique (UMR 7291), Laboratoire de Neurosciences Cognitives, Aix-Marseille Université, 13331 Marseille, France; 2 ONERA, The French Aerospace Lab, 13300 Salon de Provence, France

**Keywords:** hearing, perception, awareness, neural correlates, electroencephalogram

## Abstract

**Simple Summary:**

Characterizing the brain activity related to the conscious perceptive experience is an important step toward understanding the relationship between brain activity and consciousness. In this study, we use an experimental protocol, where a human subject detects an auditory target embedded in a multitone masker. Since the subject can miss some targets, this experimental protocol provides trials where the target is present and perceived and some trials where it is present but not perceived. Comparing the difference in brain activity between these two conditions allows us to characterize specific patterns of brain activity related to auditory perceptual awareness. Here, we provide extensive characterization of the neural correlates of auditory perceptual awareness using information-theoretic approaches in brain electrical activity together with a more conventional analysis of brain signals. Among the information measures, integrated information measures are related to a specific theory of consciousness. We show that auditory perceptual awareness is associated with an enhancement in the informational content of the neural signals in fronto-central brain areas and with an increase in the redundancy of the information in the temporal cortices. These results thus characterize conscious perceptual states on the basis of the informational content of neural signals.

**Abstract:**

In informational masking paradigms, the successful segregation between the target and masker creates auditory perceptual awareness. The dynamics of the build-up of auditory perception is based on a set of interactions between bottom–up and top–down processes that generate neuronal modifications within the brain network activity. These neural changes are studied here using event-related potentials (ERPs), entropy, and integrated information, leading to several measures applied to electroencephalogram signals. The main findings show that the auditory perceptual awareness stimulated functional activation in the fronto-temporo-parietal brain network through (i) negative temporal and positive centro-parietal ERP components; (ii) an enhanced processing of multi-information in the temporal cortex; and (iii) an increase in informational content in the fronto-central cortex. These different results provide information-based experimental evidence about the functional activation of the fronto-temporo-parietal brain network during auditory perceptual awareness.

## 1. Introduction

The extraction of an auditory target from its noisy environment is classically illustrated by the cocktail party” situation [1,2], where the processes of auditory scene analysis segregate multiple acoustic sources into coherent auditory objects [3]. These segregated objects form the basis of the listener’s conscious auditory perception, which develops gradually over time [4,5,6]. This process was experimentally studied using the masking phenomena which occur when the threshold of audibility of a given signal is raised by the presence of another sound. In particular, informational masking appears when the target is masked by a multi-tone background [7,8], whereas there is no overlap of their frequency content and no peripheral interaction at the cochlear level [9]. Awareness of the target is thus limited by information-processing bottlenecks in the central auditory system rather than by the resolution of the peripheral auditory system [10]. Successful segregation between the target and masker streams comes from information processing at a central level of the auditory system [11,12,13]. More extensive activity associated with recursive and integrative processing within a fronto-temporo-parietal brain network is also considered an essential neural substrate for auditory perceptual awareness [14,15,16,17,18,19,20]. A dynamic cascade of neural information processing based on interactions between bottom–up and top–down processes [21,22] generates modifications within the brain activity, which are referred to as neural correlates of auditory perceptual awareness. As a consequence, the quantification of the brain electrical activity during the build-up of auditory perceptual awareness may elucidate a deeper characterization of the neuronal activity related to the awareness of the auditory target [23,24,25].

Two event-related potentials (ERPs), awareness-related negativity (ARN) and the P300 (a positive component with maximum amplitude around 300 ms post-stimulus) were studied to decipher the mechanisms of information processing at the macroscopic level. ARN was observed in the auditory cortex of both hemispheres during the awareness of target tones in informational masking, with a larger amplitude for detected tones than for non-detected tones [10,19,26]. The ARN was considered a potential neural correlate of auditory perceptual awareness [10,15,16,19,26,27]. The P300, previously considered a processing marker of global stimulus integration [28,29,30,31], was larger and had a shorter latency for tones detected with high confidence than for those detected with low confidence [32,33,34]. In informational masking, the P300 was the only amplified component for detected tones, and a robust P300-like response was observed for the detected tones using trial-by-trial perceptual reports [19]. Source estimates allow one to localize P300 generators in the temporo-frontal and temporo-lateral cortices [27]. Although ERP provides information about the specific and localized processing of incoming stimuli, they do not inform about the information content of the neural signals related to perceptual awareness.

Several algorithms have been used to characterize the information content of brain electrical activity during global conscious states. Although they share the term “entropy” and thus characterize a level of disorder, they are applied to different representations of the signals and quantify different properties of the signal. Some measures are based on frequency-domain computations, like spectral entropy (SpEn) [35,36,37,38,39,40] or linear decompositions like single-value decomposition entropy (SvEn) [41,42,43]. They characterize mostly linear statistical properties of the signals, while others make probabilistic estimates in the time domain, like approximate entropy (ApEn) [38,44,45,46], sample entropy (SaEn) [38,47], or permutation entropy (PeEn) [36,38,40,46], which characterize mostly nonlinear properties. Consequently, they have different abilities to characterize the informational content of the neural signal. These entropy measures have already been used to characterize neuronal correlates of global conscious states [36,38,40,48,49]. Notably, entropy measures were employed to characterize the impaired states of consciousness like coma [50,51,52,53,54] or anesthesia [35,38,44,55,56] but also during states of consciousness modified by neuropsychiatric disorders, such as epilepsy [37,39,45,57]. Entropy measures were also used for the classification of subjective visual interest [58]. Many studies have thus shown that the informational content of the brain’s electrical activity is modulated with the state of awareness, but they have not investigated the specific changes in informational content related to the emergence of perceptual awareness.

Although entropy measures the information content of signals, Tononi et al. [59,60,61] proposed that brain activity related to consciousness would be more adequately characterized if the relationship between the integration and segregation of information in brain interactions were better taken into account. The integrated information theory of consciousness [59,60,61] was originally developed to characterize the states of consciousness. It consists of a model of consciousness that aims to establish a strong link between the properties of brain activity and the properties of conscious phenomenal experience [59,60,62]. Integrated information characterizes the difference in mutual information between the interactions in the actual system and those in a totally independent one. Several measures of integrated information were derived from this theoretical model to approach the problem of the contents of consciousness and to characterize the dynamics of the cerebral activity during perceptual awareness [63,64,65,66,67,68]. Four measures of integrated information have been developed from different theoretical points of view: (i) decoding-based integrated information [67], (ii) geometric integrated information [69], (iii) stochastic integrated information [70], and (iv) redundancy-based integrated information or “multi-information” [70]. The integrated information theory of consciousness postulates, on the one hand, that integrated information presents a relation of identity with consciousness, and on the other hand, it predicts that the measure of integrated information estimated from brain activities represents the level of consciousness. Thus, the more conscious a system, the higher its integrated information. Integrated information is considered here to study the brain’s mechanisms of information integration during auditory perceptual awareness.

We propose an exploratory study of brain electrical activity associated with auditory perceptual awareness using event-related potentials (ARN and P300), several measures of entropy, and integrated information. We present only a restricted set of related measures; a more complete characterization of these data can be found in [71]. We study the effect of target detection on the electrophysiological indices compared to non-detected targets and describe the changes in these indices over the build-up of auditory perception. The original contribution of this article is to gather different approaches, usually separated in the literature, to the electrophysiological correlates of perceptual awareness using the same data. The ARN and P300 are described as neuronal correlates of auditory awareness, and we seek to replicate the main findings in the literature. Since the information content measures were mainly used to characterize states of consciousness, we test whether they are able to quantify specific neural correlates of consciousness by distinguishing between the brain activity associated with detected targets and those related to non-detected targets. The integrated information theory of consciousness has a strong basis on the relationship between the integration of information and consciousness. In this study, we test if these hypotheses can be checked experimentally and if these measures perform better than the simple quantification of specific neural correlates of consciousness.

## 2. Materials and Methods

### 2.1. Participants

Statistical simulations were performed using the “simr” library [72] to determine the minimum sample size. In an experimental paradigm of 4 blocks with 20 statistical items each and a total number of observations higher than 1200, a sample size of n=15 subjects was considered the minimum requirement to ensure a statistical power of 84%, a medium effect size (Cohen’s *d*) =0.44, and a statistical threshold α=5%. Consequently, twenty participants (six women; ages from 20 to 39 years; mean =26 y.o., SD =4 y.o.) were recruited at the ONERA Laboratory of Salon-de-Provence following a call for volunteers. Most of the subjects (15) had an education level of 7 according to the international standard classification of education (version 2011), two had an education level of 6, and three had an education level of 8. Four subjects were left-handed (S5, S12, S13, and S19). All participants reported normal vision and filled out a general questionnaire about hearing disorders to ensure that they did not report any hearing trouble [73]. None presented neurological or psychiatric disorders or were under any medical treatment. Participants received a gift card worth EUR 30 for their contribution to the study.

### 2.2. Stimuli

**Figure 1:**
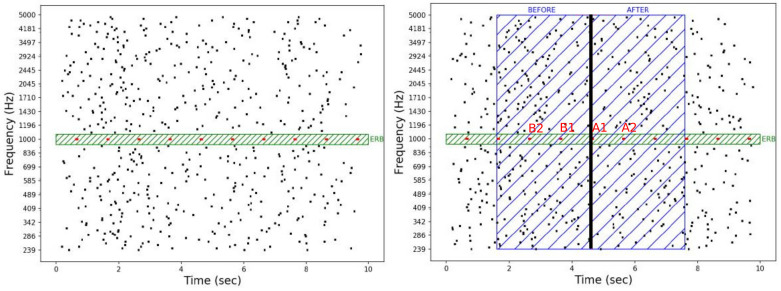
Graphical illustrations of an auditory stimulus (**left**) and epochs segmentation procedure (**right**). A target (in red) surrounded by a protected region of two equivalent rectangular bandwidths (in green) is embedded in a random multi-tone masker (in black). In these examples, the target frequency is 1 kHz with a tone duration of 60 ms and a repetition rate of 1 Hz. The masker is composed of 32 frequencies per octave with mean inter-tone intervals of 800 ms and a tone duration of 20 ms. For each trial with a target, a time reference allowed us to define a 6 s epoch (3 s before and 3 s after the time reference). For the event-related potentials analyses, four tones around the time reference were taken into account: two tones before (labeled “B1” and “B2”) and two tones after (labeled “A1” and “A2”) the time reference.

All auditory stimuli were composed of a multi-tone masker and, in two thirds of the trials, a target as depicted in Figure 1 (left). Since the acoustic parameters of the masker and the target highly influence the target detection [10,20,21,22,26,27,74,75,76], they were chosen according to a previous study [76] in order to provide a long enough delay between the beginning of the trial and the target detection, and a performance of around 70%. The target tones were presented at the same level as the individual masker tones, i.e., a target-to-masker intensity ratio of 0 dB [27].

Targets were composed of a regular series of tones defined by the tone duration (100 ms) and the tone repetition rate, i.e., tones per second (in Hz). To prevent the subject from selectively paying attention to a specific frequency range, the target tone frequencies were randomly drawn from a set of five equiprobable frequencies: 699, 1000, 1430, 2045, and 2924 Hz [10,27]. When present, the target always started at 600 ms after the beginning of the masker.

The maskers were composed of a multi-tone noise characterized by the number of frequencies per octave (fpo) and the mean inter-tone interval (miti). The ratio between the number of frequencies per octave and the mean inter-tones interval defines the spectro-temporal density of the masker (i.e., fpo/miti in s${}^{-1}$ oct${}^{-1}$). The inter-tones intervals were randomly drawn from a uniform distribution with a minimum duration of 100 ms and a variable maximum duration of either 300, 700, 1100, or 1500 ms. Tone frequencies were equally spaced on a logarithmic scale between 239 and 5000 Hz [10,20,26,27]. Each value of the mean inter-tone interval was associated with a number of frequency per octave, leading to four levels of spectro-temporal density: 11, 20, 28 and 36 s${}^{-1}$ oct${}^{-1}$. These correspond, respectively, to the pairs (miti = 200, fpo = 32), (miti = 400, fpo = 64), (miti = 600, fpo = 96), and (miti = 800, fpo = 128). All masker tones had a duration of 20 ms and included 10 ms cosine ramps.

To ensure minimal energetic masking, a protected region surrounding the target was kept free of tones in the masker. For each target, an equivalent rectangular bandwidth (ERB) [77,78] was calculated using $ERB\;=\;24.7\;(4.37\;F_{t}+1)$ with $F_{t}$ as the target frequency in kHz. The protected region was centered on the target frequency and had a total extension of two equivalent rectangular bandwidths, i.e., one on each side of the target frequency (see Figure 1). Auditory stimuli were generated using Python programming language [79]. They were digitized with a sampling rate of 44,100 Hz and a 16 audio bit depth.

### 2.3. Experimental Task and Procedure

**Figure 2:**
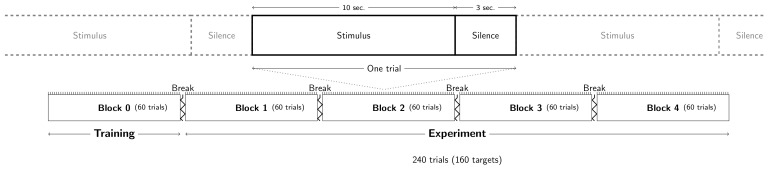
Graphical summary of the procedure. Stimuli are described in Figure 1.

A graphical summary of the procedure is presented in Figure 2.

To familiarize the participants with the experimental task and evaluate their detection rates, a training block of 60 trials was presented first. In this block, the trials were composed of maskers whose densities were sampled from the entire set of combinations of frequencies per octave and mean inter-tone interval as defined above. Targets were composed of tones with a 1 kHz frequency, a 100 ms duration, and a 1 Hz tone rate. This training block allowed us to ensure that all the subjects heard all the target frequencies.

The experimental session was composed of 240 trials randomly distributed into 4 blocks of 60 trials. The target was present in two thirds of the trials (160:240), and one third (80:240) had no target. Each trial lasted 10 s, and subsequent trials were separated by 3 s of silence. The participants’ task was to push a key press with their right index finger as soon as they detected the target (response box: Chronos Psychology Software Tools Inc., Pittsburg, PA, USA). Subjects were asked to answer as quickly and accurately as possible. They were informed that the target signal would not be present at each trial, but no information regarding the target occurrence probability was provided.

A white fixation cross was displayed against a black background onto a 19 in the cathode-ray tube monitor (with a 1024×768 pixel resolution and a 100 Hz refresh rate) located 46 cm away from the participant in a dark and soundproof room. Auditory stimulus was produced by a DELL PRECISION M4800 computer (i7 4900 MQ processor, 16 Gb DDR3 RAM, NVidia Quadro K2100M running Windows 7 with an Intel Lynx Point PCH sound card) and presented to listeners via ER-3 insert earphones (Etymotic Research) at a comfortable listening level. E-prime 2.0 software (v.2.0.10.356, E-prime Psychology Software Tools Inc., Pittsburg, PA, USA) was used to present auditory stimuli and visual fixation stimulus.

### 2.4. Behavioral Responses

The detection times were recorded each time the participant first pressed the key during a trial. Each trial was categorized as either a hit, a miss, a false alarm, or a correct rejection according to both the target’s presence and the participant’s response. Since detecting a regularity requires hearing at least two repetitions of the target tone, any detection occurring faster (i.e., before 1600 ms) was considered a guess and dismissed from the valid responses [76]. The detection performance index ($d^{\prime }$) was computed from the hit rate (HR) and false alarm rate (FAR) after a z-score transformation with the percent point function [80,81]: $d^{\prime }\;=\;z\left(\mathrm{HR}\right)-z\left(\mathrm{FAR}\right)$. The detection performance index ($d^{\prime }$) was then obtained for each level of the spectro-temporal density of the masker.

### 2.5. Electroencephalogram Recordings

**Figure 3:**
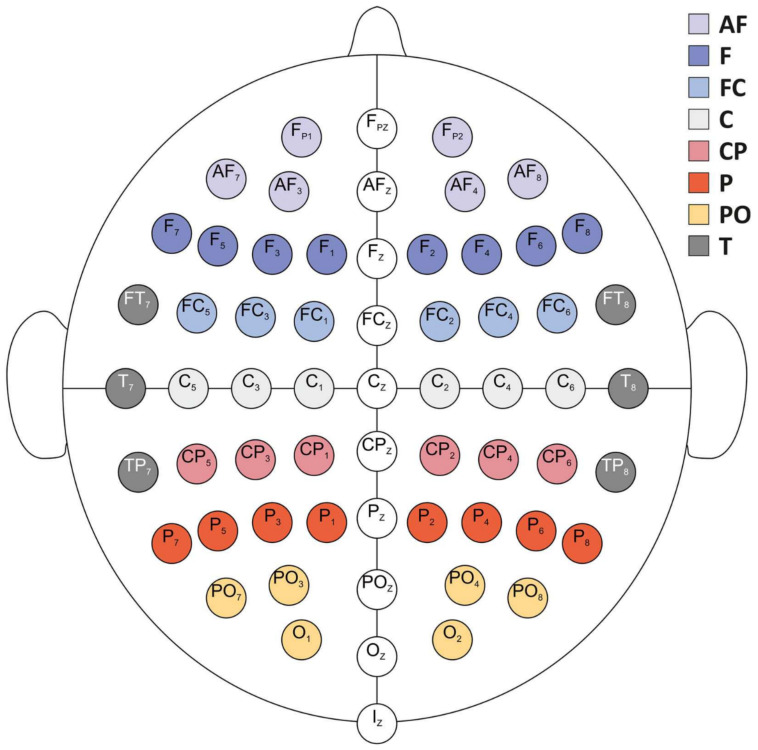
Electrode positions according to the extended 10/20 international placement system and aggregation procedure according to eight different cortical areas for each hemisphere and sagittal cluster. AF: antero-frontal; F: frontal; FC: fronto-central; C: central; CP: centro-parietal; P: parietal; PO: parieto-occipital; T: temporal. Adapted from [97].

The electroencephalogram (EEG) was continuously recorded from the head surface using a suitable elastic cap (ActiCAP, Brain Products GmbH) equipped with 64 Ag/AgCl unipolar active electrodes positioned according to a subset of the extended 10/20 electrode placement system [82,83] (Figure 3). The electrodes’ impedance was kept below 10 kΩ. The signal was amplified using an actiCHamp™ system (Brain Products, Inc.), digitized at a 24-bit rate and sampled at 1000 Hz with a 0.05 μV resolution. The raw EEG signal was recorded using Brain Vision Recording software (1.20.0801 version).

Two active electrodes (TP9 and TP10) were used to record the signals from the left and right mastoids, and their average was used as a reference. The ground electrode was positioned on the forehead (Fpz electrode). Two pure silver electro-oculography electrodes were positioned on the external side of the left and right eyes. The recorded electro-oculogram was used to detect ocular artifacts, such as blinks and eye movements. Participants were instructed to limit blinking and eye movements during the experimental session, and they were provided breaks between blocks to move their eyes and blink.

### 2.6. Processing of EEG Recordings

EEG data processing was performed using Python programming language [79] and “Python-MNE” module (v0.20.5) [84]. Raw EEG data were re-referenced offline to the average of electrodes. Low-pass (80 Hz) and high-pass (1 Hz) non-causal filters were applied.

Ocular movement artifacts were corrected using independent component analysis (20 components, 800 loops). Trials with excessive noise were either removed or repaired, depending on the number of bad channels, using the algorithm implemented in the “autoreject” Python module (v0.2.1) [85,86]. Finally, all remaining segments contaminated with muscular activity and/or non-physiological artifacts were rejected offline after a visual inspection.

A time reference corresponding to the pressing of the button for the detected targets (i.e., hit trials) and to the average detection time (3.4 s) for the non-detected targets (i.e., miss trials) was taken into account to define the 6 s EEG epochs for the analysis. For the ERP, four tones around a time reference were taken into account. The two tones before the time reference were labeled “B1” and “B2”, and the two tones after the time reference were labeled “A1” and “A2” (see Figure 1 (right)). For the entropy and integrated information measures, each epoch was divided into two segments of 3 s before and after the time reference (Figure 1 (right)).

The evoked potentials were computed in a time window from −200 to +500 ms around the four tones (B1, B2, A1, and A2) [10,19,26,27,74,87]. Then, the grand average waveforms were obtained for each electrode, for each tone, and for the detected and non-detected targets. The peak amplitude of the ARN component was computed in the 50–350 ms time interval [10] and that of the P300 component in the 250–500 ms time interval.

The information content of the neural signal was quantified using information-theoretic approaches based on five measures of entropy: spectral entropy (SpEn), approximate entropy (ApEn), sampled entropy (SaEn), permutation entropy (PeEn), and singular-value decomposition entropy (SvEn). All their explicit formulations and algorithms are presented in Appendix C.

In order to quantify the evolution of entropy during the build-up of the perceptual awareness, each 6 sec EEG epoch was divided into 24 windows. In order to ensure reliable measures for each window, EEG signals were oversampled at 4 kHz to compute entropy measures on 1000 data points for each window [88,89,90,91,92]. All information content measures were estimated using “antropy” [93] or “pyEEG” [94] Python modules.

Four measures of integrated information (Φ) were used to study the dynamics of auditory perceptual awareness: (i) decoding-based integrated information (Φ*) [67], (ii) geometric integrated information ($\Phi^{G}$) [69], (iii) stochastic integrated information ($\Phi^{H}$) [70], and (iv) redundancy-based integrated information, or “multi-information” ($\Phi^{MI}$) [70]. The algorithms allowing the calculation of these measures are available in the Matlab toolbox “phitoolbox” [67,68]. More complete formulations of these measures are described in Appendix D.

Since computing integrated information measures on real neurophysiological data is known to be extremely time consuming as the number of electrodes increases [68,95,96], the integrated information measures were computed for the temporal cluster only (see Figure 3) on windows of 750 time samples after the EEG signal was downsampled at 125 Hz. By varying the time lag τ involved in the computation of integrated information measures, 65 windows (27 before and 37 after the time reference) were obtained as the average of 10 τ values. In this configuration, in total, more than 3400 h using the computing cluster of ONERA were needed for this study.

### 2.7. Statistical Analyses

Information content and integrated information measures were topographically aggregated by averaging the values obtained for groups of electrodes [97]. Eight cortical areas were defined per hemisphere for the entropy measures, and only the temporal cluster was studied using the integrated information measures (see Figure 3).

The effects of the experimental conditions on behavioral data, ERP amplitude, EEG information content, and integrated information were analyzed using linear mixed-effect models implemented in the “lme” [98] and “nlme” [99] libraries in the R statistical language [100]. Five factors were used in this study: detection (hit/miss), condition (before/after the time reference), cluster (AF, F, FC, C, CP, P, PO, T, S), window (11 before and 11 after for information content measures; 27 before, 38 after for integrated information), and subject (20). The window factor is nested in the condition factor. In all analyses, the experimental factors (i.e., detection, condition, cluster, and window) were treated as fixed-effect variables, and the subject factor was treated as a random effect for the intercept.

In each case, analyses of variance were performed to test the null model (i.e., without mixed effects) against the corresponding mixed-effects model, and all of the models were checked on the basis of their residuals. The linear mixed-effects models were fitted and estimated using likelihood maximization optimizers. In the case of statistical effects of factors and their interactions, we studied the paired comparisons using the estimated marginal means [101] implemented in the R “emmeans” library. The estimated marginal means (least squares means) are the values of the model parameters averaged over the appropriate combinations of the levels of factors.

For the behavioral data, a model was adjusted to obtain the effect of the spectro-temporal density of the masker on the detection performance index. ERPs were analyzed in the function of the electrodes of interest in order to study the effects of detection and tone factors on the peak amplitude of ARN and P300 components. For the information content measures, a first model was fitted in order to study the effects of the detection, condition, and cluster factors on the measures. This allowed us to investigate the effect of the auditory perceptual awareness on the different measures before and after the time reference and within the different brain areas of interest. A second model was fitted to study the temporal dynamics of the build-up of auditory perception within the significant clusters. For the integrated information measures, a first model was fitted in order to study the effects of the detection and condition factors on the Φ measure values. Then, a second model was fitted to study the temporal dynamics of the integration of the information underlying the build-up of auditory perception.

## 3. Results

### 3.1. Behavioral Data

The individual mean detection times and performance indices for each subject are presented in Table A1. The group mean detection time was 3.45±1.58 s. The mean correct detection and false alarm rates were 0.71%±0.14% and 0.11%±0.14%, respectively, leading to a mean detection performance index ($d^{\prime }$) of 2.15±0.84. Left-handed subjects did not perform worse than right-handed subjects (all $d^{\prime }$ higher than the lower limit of the 95 % confidence interval), although one of them had a longer detection time (S12).

**Figure 4:**
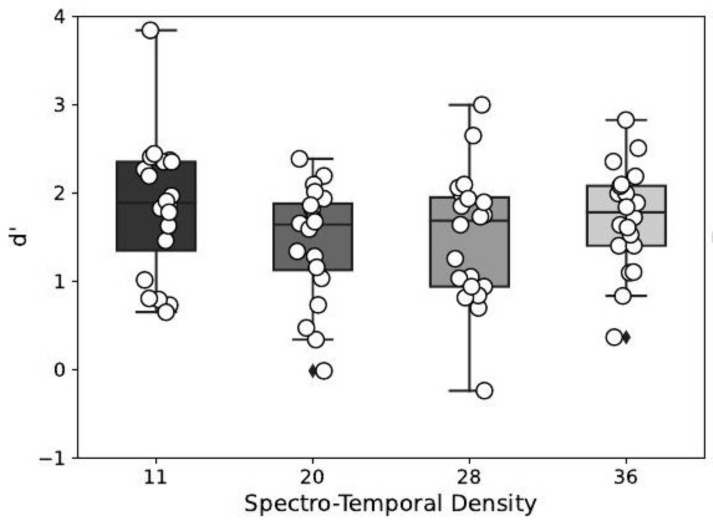
Detection performance index $d^{\prime }$ in function of the spectro-temporal density of the masker.

The effect of the masker’s spectro-temporal density on the detection performance index is depicted in Figure 4. This effect was statistically significant (F(3,57)=4.46,p=0.01), and post hoc tests show that the detection performance index was significantly lower for a masker density of 20 and 28, compared to a density of 11. No other significant effect was found for the other multiple comparisons (see Table 1).

### 3.2. Electrophysiological Data

#### 3.2.1. Event-Related Potentials ARN and P300

**Figure A1:**
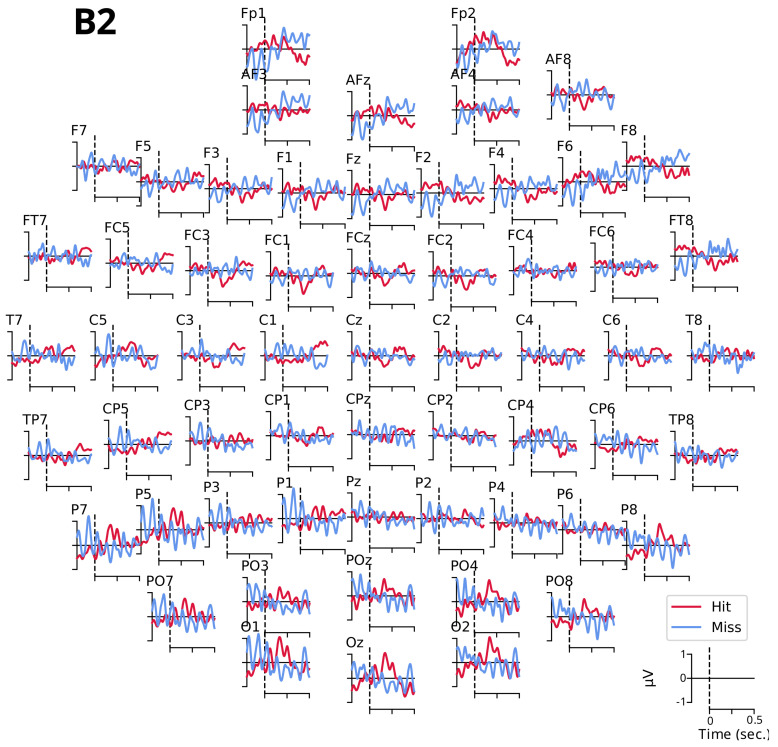
Compared grand averages of evoked potentials between −200 and +500 ms for tone B2 for detected (Hit, in red) and undetected (Miss, in blue) targets.

**Figure A2:**
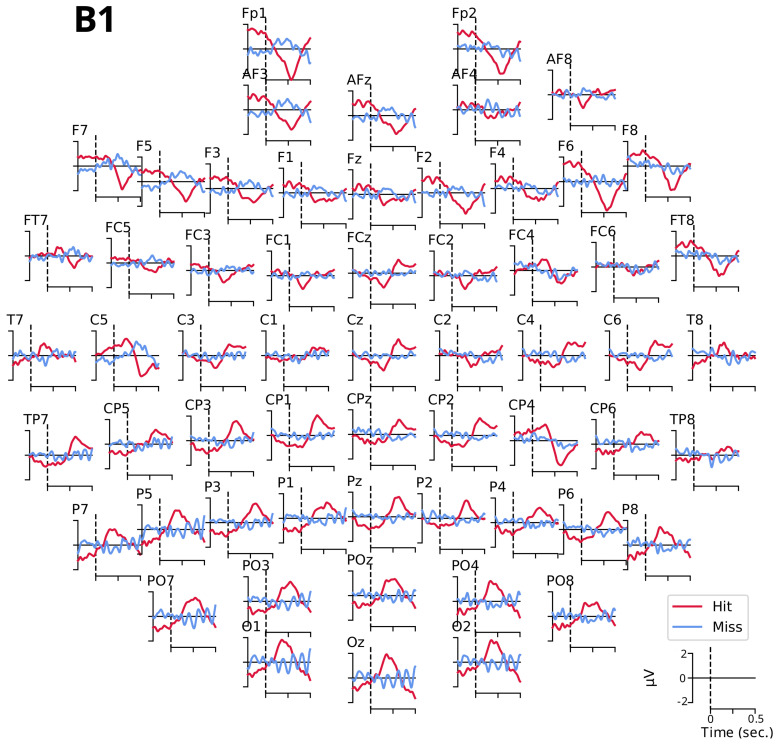
Compared grand averages of evoked potentials between −200 and +500 ms for tone B1 for detected (Hit, in red) and undetected (Miss, in blue) targets.

**Figure A3:**
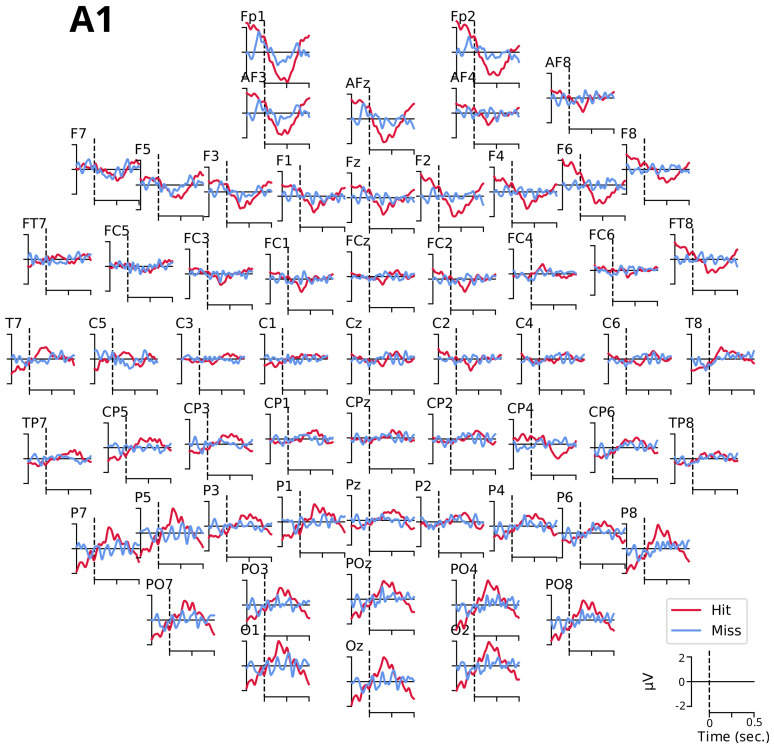
Compared grand averages of evoked potentials between −200 and +500 ms for tone A1 for detected (Hit, in red) and undetected (Miss, in blue) targets.

**Figure A4:**
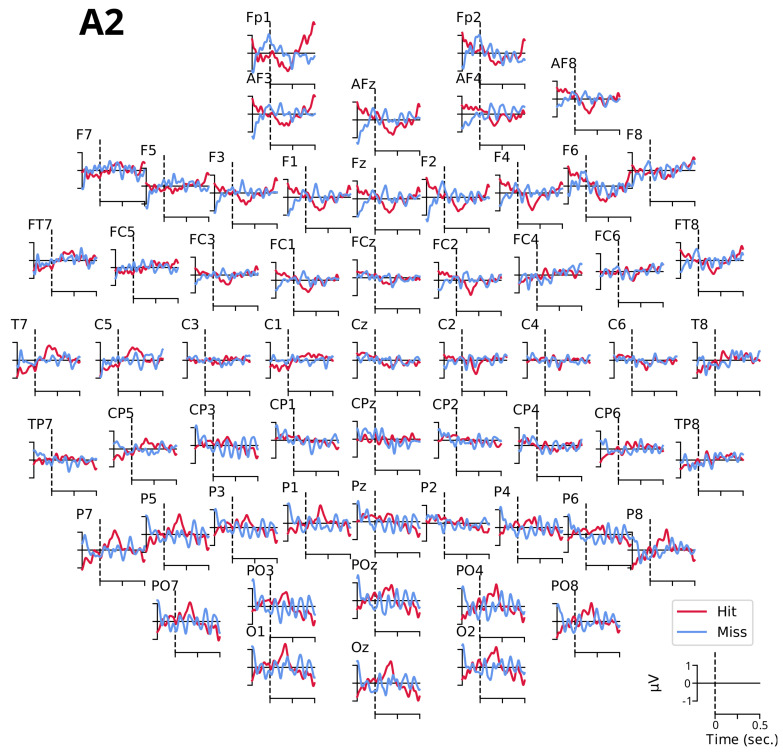
Compared grand averages of evoked potentials between −200 and +500 ms for tone A2 for detected (Hit, in red) and undetected (Miss, in blue) targets.

The complete topography of the ERP can be observed for the four tones on Figure A1, Figure A2, Figure A3 and Figure A4.

**Figure 5:**
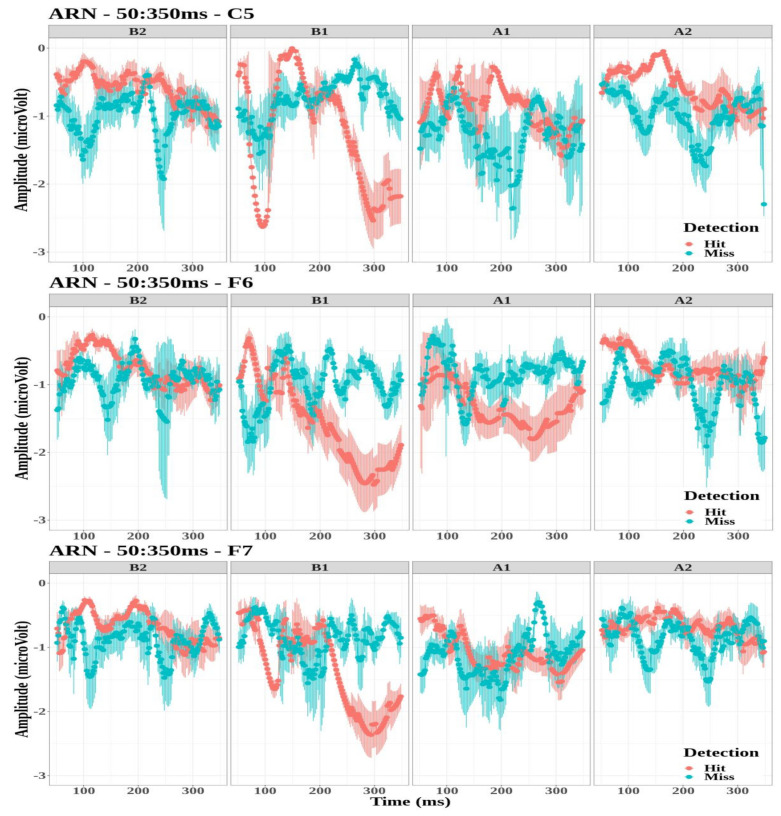
Grand-average time-evoked waveforms of the awareness-related negativity (ARN) components on electrodes C5 (**top** panel), F6 (**middle** panel), and F7 (**bottom** panel) for the first and second tone before (**B1**,**B2**) and after (**A1**,**A2**) the time reference between 50 and 350 ms.

The amplitude values of the ARN were selected for the electrodes FT7, FT8, T7, T8, TP7, TP8, C5, F6, and F7 for all tones (B2, B1, A1, and A2). The largest negative amplitudes were found for electrodes C5, F6, and F7 for the first tone before the button press (B1) for the detected targets. Figure 5 illustrates the grand-average waveforms of the ARN for electrodes C5, F6, and F7 for the four tones. Thus, the effect of detection and tone factors on peak amplitude values was studied for each electrode.

- The ARN amplitude did not differ significantly between detection conditions for the three electrodes (C5: F(1,81)=0.69, p=0.41; F6: F(1,95)=3.47, p=0.07; and F7: F(1,90)=2.74, p=0.1).
- However, the tone factor had a significant effect on the ARN amplitude for the F6 and F7 electrodes (F(3,95)=3.81, p=0.01, $\eta^{2}=0.11$ and F(3,90)=2.99, p=0.04, $\eta^{2}=0.9$, respectively), while no significant effect was observed for the C5 electrode (F(3,81)=1.76, p=0.16).
- The interaction between the detection and the tone factors was significant for the three electrodes (C5: F(3,81)=4.54, p=0.01, $\eta^{2}=0.14$; F6: F(3,95)=8.12, p<0.001, $\eta^{2}=0.2$; and F7: F(3,90)=5.05, p<0.001, $\eta^{2}=0.14$).

Multiple comparisons were performed to study the interaction between detection and tone factors (see Table 2). The amplitude of ARN was significantly lower for tone B1 when targets were detected compared to missed targets for the three electrodes. On the contrary, amplitude values were significantly higher for tone A2 when targets are detected compared to missed targets for electrode F6.

**Figure 6:**
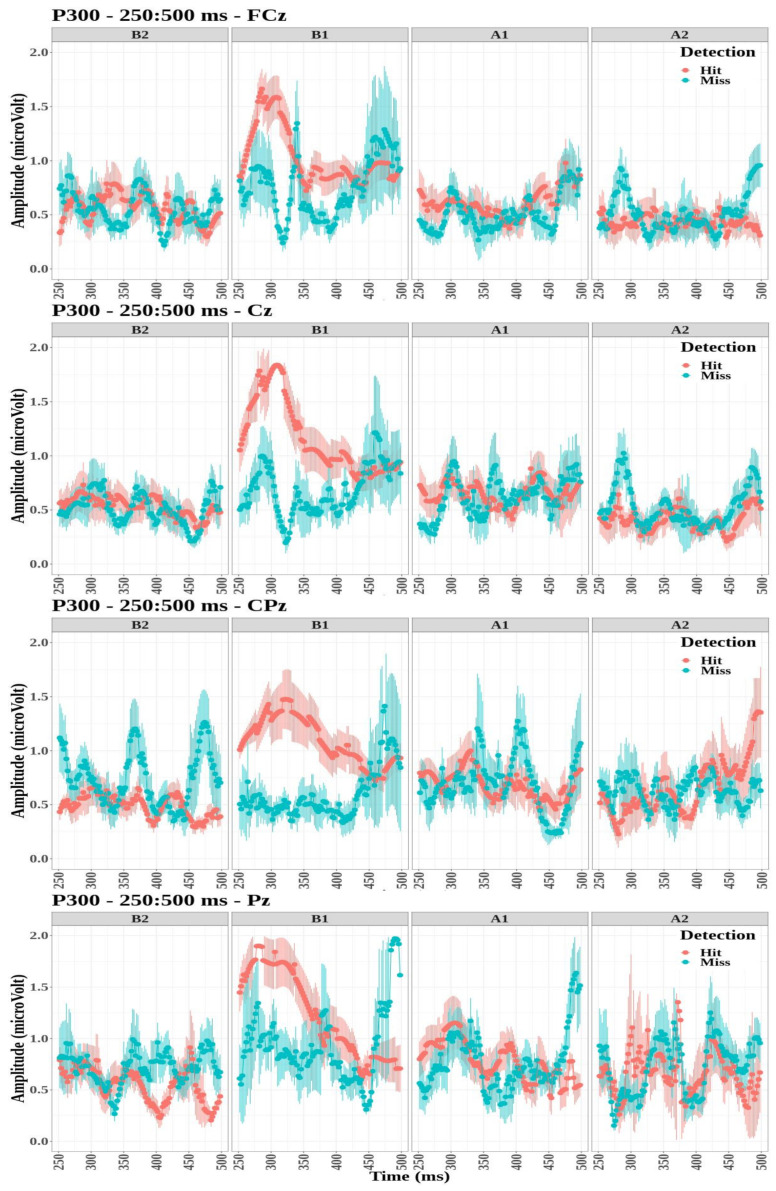
Grand-average time-evoked waveforms of the P300 components on electrodes FCz (**top** panel), Cz (**second** panel), CPz (**third** panel), and Pz (**bottom** panel) for the first and second tones before (**B1**,**B2**) and after (**A1**,**A2**) time reference between 250 and 500 ms.

The P300 component was observed in the sagittal cluster through electrodes FCz, Cz, CPz, and Pz (Figure 6). Compared evoked waveforms (see Figure A1, Figure A2, Figure A3 and Figure A4) show that the FCz, Cz, CPz, and Pz electrodes notably displayed a P300 signature with a strong transition when the targets were detected for the first tone, B1. However, this waveform was not observed for the detected targets on the other tones. For each electrode, the effect of detection and tone factors on the peak amplitude was studied.

- The P300 amplitude did not differ significantly between detection conditions for the four electrodes (FCz: F(1,101)=0.14, p=0.71; Cz: F(1,102)=0.35, p=0.55; CPz: F(1,99)=0.13, p=0.71; and Pz: F(1,101)=0.95, p=0.33).
- The tone factor had a significant effect on the amplitude of P300 for the FCz, Cz, and Pz electrodes (F(3,101)=10.3, p<0.001, $\eta^{2}=0.23$; F(3,102)=8.02, p<0.001, $\eta^{2}=0.19$; and F(3,101)=6.75, p<0.001, $\eta^{2}=0.17$, respectively) but not for the CPz electrode (F(3,99)=0.92, p=0.43).
- The interaction between the detection and the tone factors was significant for all four electrodes (FCz: F(3,101)=7.14, p<0.001, $\eta^{2}=0.17$; Cz: F(3,102)=8.06, p<0.001, $\eta^{2}=0.19$; CPz: F(3,99)=11.21, p<0.001, $\eta^{2}=0.25$; and Pz: F(3,101)=9.54, p<0.001, $\eta^{2}=0.22$).

Multiple comparisons were then performed to study the interaction between detection and tone factors (see Table 2). The amplitude of P300 was significantly higher for tone B1 when targets were detected compared to missed targets for the all electrodes. The amplitude of P300 was significantly lower for tone B2 when targets were detected compared to missed targets for the CPz electrode. Finally, the amplitude values were significantly lower for tone A2 when targets were detected compared to missed targets for electrodes FCz, Cz, and Pz.

#### 3.2.2. Information Content Measures

**Figure 7:**
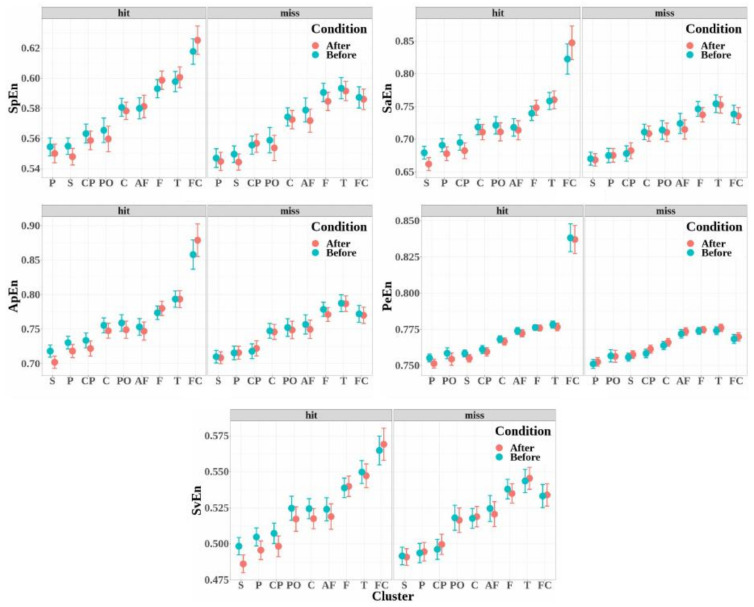
Mean values and standard errors of the entropy measures calculated for each of the clusters (AF: antero-frontal, F: frontal, FC: fronto-central, C: central, CP: centro-parietal, P: parietal, PO: parieto-occipital, T: temporal, and S: sagittal) before and after target detection (hit, in light red) or non-detection (miss, in light blue). Measures were computed from each electrode in the cluster and then obtained by topographic aggregation of the values.

The mean values of the five entropy measures for each electrode cluster are depicted in Figure 7 in increasing order. The variations in the entropy according to the experimental factors (detection, condition, and electrodes clusters) were similar for the five entropy measures:

- The entropies obtained for the electrode clusters differed significantly for the five entropy measures (SpEn: F(8,647)=51.21, p<0.001, $\eta^{2}=0.39$; ApEn: F(8,647)=49.65, p<0.001, $\eta^{2}=0.38$; SaEn: F(8,647)=46.06, p<0.001, $\eta^{2}=0.36$; PeEn: F(8,647)=147.72, p<0.001, $\eta^{2}=0.65$; and SvEn: F(8,647)=46.04, p<0.001, $\eta^{2}=0.36$). Moreover, the four electrode clusters that depicted the highest entropy values were the same for all the entropy measures, and entropy increased from the antero-frontal cluster to the fronto-central cluster through the frontal and temporal cluster.
- The entropy measures were significantly higher when the target was detected (hits) than when the target was not detected (miss), except for the singular-value entropy (SpEn: F(1,647)=6.64, p=0.01, $\eta^{2}=0.01$; ApEn: F(1,647)=7.94, p=0.005, $\eta^{2}=0.01$; SaEn: F(1,647)=5.54, p=0.019, $\eta^{2}=8.49\times 10^{-3}$; PeEn: F(1,647)=91.16, p<0.001, $\eta^{2}=0.12$; and SvEn: F(1,647)=1.65, p=0.19).
- No significant effect was observed for the condition factor for all the entropy measures (SpEn: F(1,647)=1.09, p=0.297; ApEn: F(1,647)=0.84, p<0.358; SaEn: F(1,647)=0.68, p<0.408; PeEn: F(1,647)=0.12, p<0.725; and SvEn: F(1,647)=1.63, p=0.201).
- The interaction of the detection factor and cluster was significant for all the entropy measures (SpEn: F(8,647)=2.82, p=0.004, $\eta^{2}=0.03$; ApEn: F(8,647)=9.61, p<0.001, $\eta^{2}=0.11$; SaEn: F(8,647)=7.88, p<0.001, $\eta^{2}=0.09$; PeEn: F(8,647)=76.56, p<0.001, $\eta^{2}=0.49$; and SvEn: F(8,647)=2.70, p=0.006, $\eta^{2}=0.03$).
- The effect of the interaction between detection and condition was significant for the permutation entropy only (SpEn: F(1,647)=0.42, p=0.51; ApEn: F(1,647)=0.08, p=0.77; SaEn: F(1,647)=0.00, p=0.95; PeEn: F(1,647)=4.53, p=0.034, $\eta^{2}=6.97\times 10^{-3}$; and SvEn: F(1,647)=1.48, p=0.22).
- The effect of the interaction between condition and cluster was not significant (SpEn: F(8,647)=0.25, p=0.98; ApEn: F(8,647)=0.29, p=0.96; SaEn: F(8,647)=0.29, p=0.96; PeEn: F(8,647)=0.13, p=0.99; and SvEn: F(8,647)=0.18, p=0.99).
- The effect of the triple interaction between detection, condition, and electrode cluster factors was not significant (SpEn: F(8,647)=0.26, p=0.97; ApEn: F(8,647)=0.42, p=0.90; SaEn: F(8,647)=0.46, p=0.88; PeEn: F(8,647)=0.06, p=1.00; and SvEn: F(8,647)=0.24, p=0.98).

Multiple comparisons were performed to study the interaction between detection and cluster factors for all the entropy measures (see Table 3). In the fronto-central cluster, entropy measures increased significantly for the detected targets compared to missed targets for all the entropy measures. In the other clusters, except for the central and temporal clusters, the permutation entropy only depicted a significant difference between the detected and non-detected targets. This difference corresponds to a decrease in entropy for the detected targets compared to the non-detected ones.

**Figure 8:**
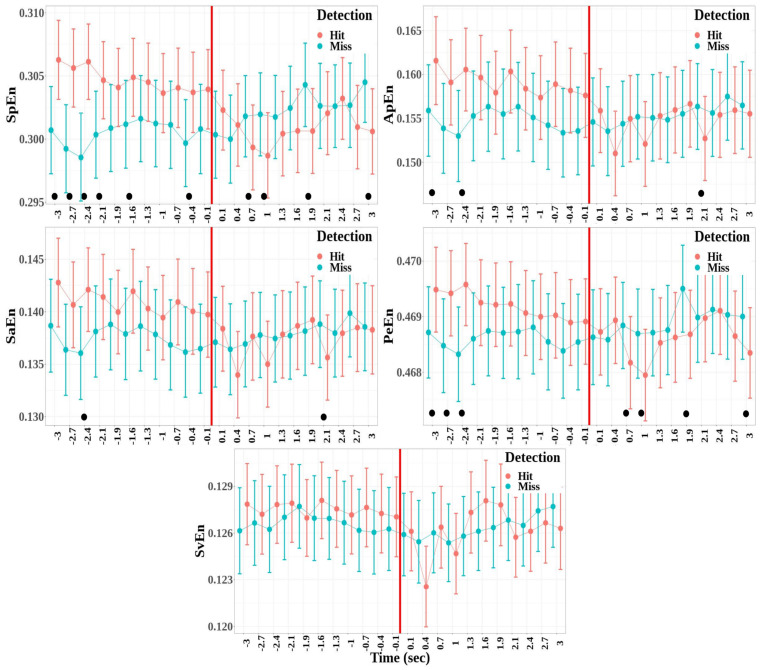
Mean values and standard error bars of entropy measures computed for each of the time windows from −3 to +3 s, respectively, before and after the time reference (red line) for the fronto-central cluster. The values of the measures were estimated from 1000 point windows. The black dots show the time windows where a significant difference was found in the model between detection and non-detection.

The dynamics of the entropy measures was studied for the fronto-central cluster, which depicts significant differences between the detected and non-detected targets for all the entropy measures (see Figure 8). The effects of the detection and windows factors are the following:

- There was no significant difference between the hit and miss trials for all the entropy measures (SpEn: F(1,795)=3.43, p=0.06; ApEn: F(1,795)=2.58, p=0.10; SaEn: F(1,795)=1.33, p=0.24; PeEn: F(1,795)=0.60, p=0.43; and SvEn: F(1,795)=2.79, p=0.09)
- A significant effect of the window factor was found for the approximate, the sample, and the singular-value entropies, but not for the spectral and permutation entropies (SpEn: F(21,795)=1.39, p=0.11; ApEn: F(21,795)=1.66, p=0.031, $\eta^{2}=0.04$; SaEn: F(21,795)=1.60, p=0.044, $\eta^{2}=0.04$; PeEn: F(21,795)=1.42, p=0.09; and SvEn: F(21,795)=1.63, p=0.038, $\eta^{2}=0.04$).
- A significant effect was also reported for the interaction between detection and window for all the entropy measures except for the singular-value decomposition entropy (SpEn: F(21,795)=5.22, p<0.001, $\eta^{2}=0.12$; ApEn: F(21,795)=1.90, p=0.009, $\eta^{2}=0.05$; SaEn: F(21,795)=1.68, p=0.028, $\eta^{2}=0.04$; PeEn: F(21,795)=2.76, p<0.001, $\eta^{2}=0.07$; and SvEn: F(21,795)=0.92, p=0.569).

Then, multiple comparisons were performed between hit and miss trials for each time window for entropy measures, where the interaction between the detection and time window was significant (SpEn, ApEn, SaEn, and PeEn). The time windows where the difference was statistically significant are represented by black dots in Figure 8. Several time windows depict a significant difference between hit and miss trials. The general pattern, which was the most visible for spectrum and permutation entropies, shows high entropy before the time reference for the hit trials compared to the miss trials and a decrease in entropy in the windows following the time reference. Although there was no significant interaction between the detection and time window for singular-value entropy, this pattern was also qualitatively present for this measure.

#### 3.2.3. Integrated Information Measures

**Figure 9:**
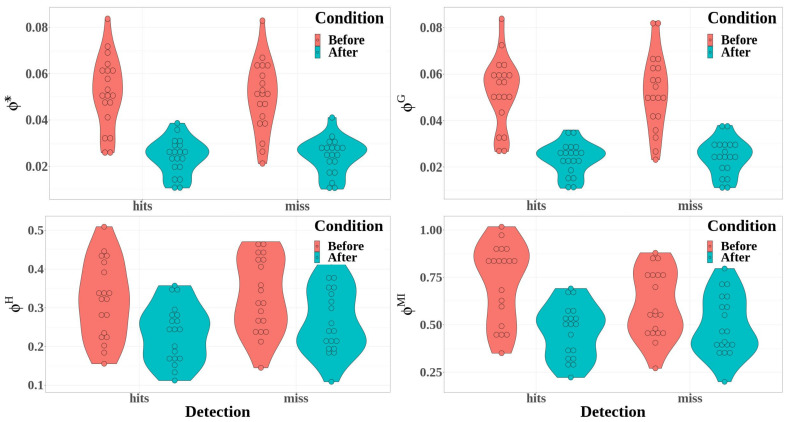
Integrated information measures computed from the temporal cluster are represented here as a function of the detection (detected or missed targets) and the condition (before, in red; after, in blue): Φ* (**top left**); $\Phi^{G}$ (**top right**); $\Phi^{H}$ (**bottom left**); and $\Phi^{MI}$ (**bottom right**).

Figure 9 shows the distribution of the values of the four integrated information measures as a function of detection (detected or missed targets) and condition (before or after the detection).

- The difference between hit and miss trials was significant for stochastic integrated information and multi-information, whereas it was not significant for the decoding-based and geometric integrated information (Φ*: F(1,54)=0.51, p=0.479; $\Phi^{G}$: F(1,54)=0.03, p=0.859; $\Phi^{H}$: F(1,54)=4.08, p=0.048, $\eta^{2}=0.07$; $\Phi^{MI}$: F(1,54)=4.29, p=0.043, $\eta^{2}=0.07$)
- Integrated information decreased significantly between before and after the time reference for the four measures of integrated information (Φ*: F(1,54)=580.20, p<0.001, $\eta^{2}=0.91$; $\Phi^{G}$: F(1,54)=541.93, p<0.001, $\eta^{2}=0.91$; $\Phi^{H}$: F(1,54)=49.39, p<0.001, $\eta^{2}=0.48$; and $\Phi^{MI}$: F(1,54)=69.34, p<0.001, $\eta^{2}=0.56$).
- The interaction between detection and condition was significant for the multi-information only (Φ*: F(1,54)=0.75, p=0.390; $\Phi^{G}$: F(1,54)=0.15, p=0.698; $\Phi^{H}$: F(1,54)=1.01, p=0.319; and $\Phi^{MI}$: F(1,54)=11.03, p=0.002, $\eta^{2}=0.17$).

Multiple comparisons allowed us to study the interaction between condition and detection for multi-information measures. They show that, before the time reference, $\Phi^{MI}$ was significantly higher for hit trials than for miss trials ($t_{54}=3.813$, p<0.001), but this difference was no more significant after the time reference ($t_{54}\;=\;-0.884$, p=0.380).

**Figure 10:**
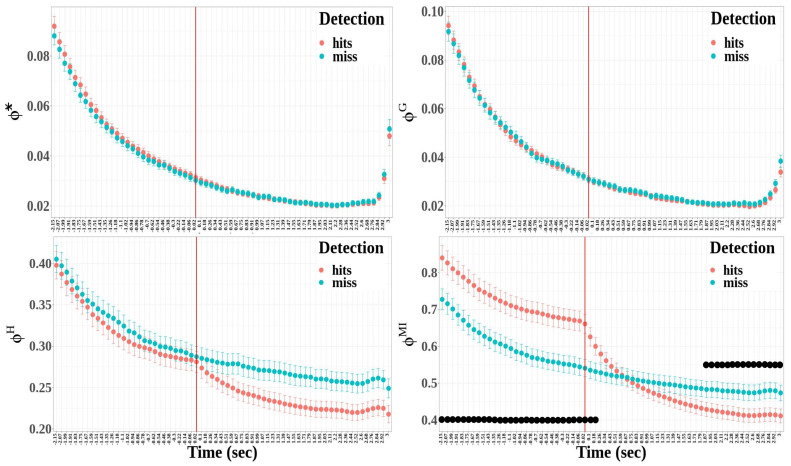
Time evolution of integrated information measures around the time reference. The measures appear as a function of τ and detection in the following order: Φ*, $\Phi^{G}$, $\Phi^{H}$ and $\Phi^{MI}$. The red vertical bars represent the time reference. The black dots show the time windows expressing a statistically significant difference between detected and missed targets.

The changes in the integrated information with the time delay τ are represented in Figure 10 for hit and miss trials. The effects of the detection and time window factors are the following:

- Except for the geometric integrated information, the integrated information significantly differed between hit and miss trials (Φ*: F(1,2322)=7.58, p=0.006, $\eta^{2}=3.25\times 10^{-3}$) $\Phi^{G}$: F(1,2322)=1.38, p=0.241; $\Phi^{H}$: F(1,2322)=187.54, p<0.001, $\eta^{2}=0.07$; and $\Phi^{MI}$: F(1,2322)=98.95, p<0.001, $\eta^{2}=0.04$).
- The time window factor had a significant effect for the four integrated information measures (Φ*: F(64,2322)=356.27, p<0.001, $\eta^{2}=0.91$; $\Phi^{G}$: F(64,2322)=331.52, p<0.001, $\eta^{2}=0.90$; $\Phi^{H}$: F(64,2322)=42.46, p<0.001, $\eta^{2}=0.54$; and $\Phi^{MI}$: F(64,2322)=55.38, p<0.001, $\eta^{2}=0.60$).
- The effect of the interaction between detection and time windows was significant only for the multi-information (Φ*: F(64,2322)=0.46, p>0.999; $\Phi^{G}$: F(64,2322)=0.28, p>0.999; $\Phi^{H}$: F(64,2322)=0.79, p=0.892; and $\Phi^{MI}$: F(64,2322)=8.54, p<0.001, $\eta^{2}=0.19$).

Multiple comparisons were performed between hit and miss trials for each time window for multi-information, where the interaction between detection and time window was significant. The time windows where the difference was statistically significant are represented by black dots in Figure 10. The statistical differences demonstrate that integrated information was higher for hit trials than for miss trials at the beginning of the 6 s epoch, whereas it was lower at the end of the epoch.

## 4. Discussion

The aim of this study was to characterize the neural correlates of auditory awareness using event-related potentials, information-theoretic measures, and measures derived from the integrated information theory of consciousness. The comparisons of the informational measures between hit and miss trials, before and after the time reference of the target perception combined with a topographic aggregation of electrodes cluster, provide the basis of our results.

The combinations of masker parameters (frequencies per octave and mean inter-tone intervals) used here allowed us to observe a long enough time period for the build-up of the auditory perception and sufficient hit trials. Obtaining information and integrated information measures was thus highly dependent on this specific set of masker parameters. Therefore, characterizing the behavioral effect of different masker density levels is crucial for the design of experimental paradigms adapted to the study of the neuronal correlates of perceptual auditory awareness [76]. Despite its relatively homogeneous spectro-temporal density, a significant effect of the masker density was observed. Indeed, the interaction between the spectral and temporal characteristics of the masker can highly modulate the segregation of the target from the noisy background masker [10,22,76]. Since the lowest masker density was associated with high detection performance, the masker density may be an index of the detectability level. However, since no monotonic relationship was observed between the detection performance and masker density, and since the masker and target properties highly interact in informational masking [76], it remains difficult to define the target and masker parameters to ensure a defined level of detectability.

In the present study, a waveform similar to the ARN was observed when the target was detected for the first tone (B1) preceding the perceptual report but not for the second one (B2). This negativity localized near the right and left temporal lobes appeared between 250 and 350 ms and exhibited characteristics similar to those of an ARN. In a comparable masking protocol, Gutschalk et al. [10] observed an ARN two tones before the report, which corresponds here to the B2 tone. In a protocol using pairs of tones as targets [19], the ARN appeared just before the perceptual report by the subject, and thus corresponded to tone B1 in our study. In the case of targets with four tones [26], the ARN was observed for the second, third, and fourth tones, while the first tone showed latency characteristics different from those of the other three. The amplitude of this negative wave was maximal in the most anterior sites of the scalp, which is consistent with the literature [102]. Dynamical causal modeling analysis [19] suggests that ARN would characterize the auditory stream segregation by being associated with changes in the intrinsic connectivity of auditory cortices. The ARN response observed here can thus be considered a signature of the auditory perceptual awareness associated with recurrent processing between higher-order auditory and parietal cortical areas.

A tone-dependent effect of auditory perceptual awareness was observed on the amplitude of a P300-like waveform for all the electrodes of interest. This effect increased significantly for the first tone before detection. Giani et al. [19] observed that perceptual awareness significantly increased P300 amplitudes for both tones for a target made of a pair of tones. A large P300 observed for the detected targets was associated with generators in the temporo-frontal and temporo-lateral cortices [27]. In the present study, large amplitudes of P300 were observed at the level of the sagittal axis. P300 is considered a marker of the segregation of the auditory target’s stream from the masker stream [19,27]. The P300 wave is composed of at least two distinct subcomponents, an early fronto-central P3a and a later maximal parietal P3b [103]. P3a occurs in states of unawareness and reflects automatic, stimulus-driven attention processes, such as when an unexpected stimulus involuntarily draws attention [104]. P3b, on the other hand, is most often elicited in experimental settings during tasks involving infrequent target detection and is thought to reflect the working memory storage of content, stimulus–response transformations, context updating, stimulus categorization, and perceptual awareness [31,103,104]. This could suggest that the P300 is related to the integration of target features, which is therefore observed by an increase in its amplitude on the vertex as the subject tends towards the report. However, recent studies contradict the role of P3b in perceptual awareness and tend to associate this ERP component rather with the post-perceptual process than with the conscious integration process [105,106,107,108,109].

Most of the entropy measures depict similar results, where entropy values are higher for the hit trials than for the miss trials, and identify the fronto-central cluster as the area where this difference is the most clearly detected. Nevertheless, the permutation entropy is the only measure to show both higher values in the fronto-central cluster and lower values in most other clusters during the auditory perceptual awareness. In previous studies, permutation entropy and other nonlinear measures, such as approximate entropy, were shown to better distinguish EEG in conscious states from EEG in unconscious states and to better reflect different levels of general anesthesia [38,55,88]. Higher permutation entropy in non-vegetative subjects than in vegetative subjects was also found in centro-posterior brain areas [40]. These results are associated with the ability of permutation entropy to detect non-linearities where linear measures, such as spectral entropy, fail [110,111,112]. High permutation entropy in the fronto-central cluster during target awareness indicates a decrease in the predictability of EEG signals, which could be due to growths in the signal fluctuations, while the opposite is observed at the level of the other clusters, where lower permutation entropy is observed for perceived targets. This difference between fronto-central areas and other brain areas might characterize their differential involvement in discriminative ability and perceptual awareness.

The evolution of the entropy measures over the time course of the trial shows high entropy measures prior to the time reference for the detected targets. The values then return to the level of the undetected targets after the time reference. This result suggests a higher information content at the scale of the neural signal during the build-up of the conscious perception and a decrease in the informational content once the target has been perceived as shown by the significant difference in the windows following the time reference for permutation entropy. Indeed, the decrease in entropy in the fronto-central brain areas following the time reference only when the targets have been detected could be related to report initialization and decision making [109,113] and be linked to specific post-perceptual processing aspects [103,107]. Specifically, it might suggest that once the processing associated with the stimulus is performed, and therefore that the perceptual report is carried out, the sudden decrease in the informational content of the neural signal is associated with a kind of reset of the information processing. Such a hypothesis requires further examination to evaluate its consistency and generality.

The results for the integrated information measures are less uniform than in the case of the entropy measures. Although all the integrated information allows us to discriminate between before and after the time reference, multi-information only depicts a significant interaction between condition and detection. Moreover, multi-information only depicts a significant time evolution during the time course of the trials. Focusing on the results obtained for multi-information, we can describe the changes in integrated information during auditory perceptual awareness. First, multi-information was globally higher for hit trials than for miss trials in temporal brain areas, and windows with higher multi-information also characterized hit trials before the time reference. A progressive decrease in multi-information was observed during the build-up of the auditory perception. Then, a clear drop appeared after the behavioral report, leading to lower multi-information for the hit trials than for the miss trials at the end of the epoch. As the multi-information is intended to capture the integration of information by measuring the amount of information shared in the connected system compared to the system where all interactions are removed [70], the build-up of auditory perceptual awareness is characterized in the temporal cluster by the evolution of brain activity from highly integrated information processing before the behavioral report to more independent processes after the behavioral report.

The characterization of the neural correlates of auditory perceptual awareness by permutation entropy and multi-information provides a complementary description of EEG information content and integration in both space and time. In informational masking, the process of segregating the auditory streams leading to the awareness of the auditory target is based on a cascade of information processing [10,27], taking just a few hundred milliseconds to activate different brain regions. Previously, it was suggested that auditory perceptual awareness is underpinned by recurrent processes in the auditory cortex [19], and our results show that these recurrent processes may correspond to a high level of integrated information in the temporal cluster as demonstrated by the multi-information.

Moreover, the planum temporale, located on the superior temporal gyrus and posterior to Heschl’s gyrus [114], has is considered an efficient neural center for encoding the statistical properties of acoustic signals [115]. An efficient encoding mechanism takes place using fewer computational resources when less information is present in the auditory stimulus, suggesting that the planum temporale is a functional center requiring fewer computational resources to encode redundant signals compared to those with high entropy [115]. These results might be supported by the high entropies for hit trials in the fronto-central cluster, whereas they do not differ from miss trials for central and temporal clusters and are characterized by low entropies in the other clusters. Further examination should focus on this link between the information content of the auditory stimulus and that of neural signals.

Finally, as auditory scene analysis involves analyzing a mixture of sounds to elicit perceptions that correspond to individual sound sources [3], such processes are reminiscent of the way in which information accumulates over time at the auditory system level. As the auditory system is able to store acoustic information in the short-term memory and make available the stored information for recall and computation in the longer term [116], it would be highly likely that a successful segregation is dependent on the accumulation of statistical evidence based on acoustic features [117,118]. If evidence accumulation processes of auditory information are handled by the core regions of the auditory cortex, once a sufficient amount of information has been accumulated, the decision limit can be exceeded, triggering the perceptual report. The observed decrease in the multi-information might be a consequence of the implementation of such mechanisms within the auditory cortices, whose role is to maintain the consistency and the coherency of the acoustic information over time until the accumulated evidence is sufficient to solve the uncertainty of the stimulus and consequently support the perceptual segregation between the masker and the target.

## 5. Conclusions

This study provides an extensive characterization of the neural correlates of auditory perceptual awareness using information-theoretic approaches in EEG together with ERP analysis. The ARN and P300 components described here are comparable to those found in the previous literature, ensuring that the informational quantification undertaken on the same data is relevant regarding the neuronal correlates of the auditory perceptual awareness. Among the entropy measures, permutation entropy showed the most complete characterization of the manifold modifications of brain electrical activity related to auditory perceptual awareness. The integrated information quantified by multi-information was higher for perceived targets than for non-perceived ones, in accordance with the hypothesis of the integrated information theory of consciousness. Taken as a whole, permutation entropy and multi-information seem to provide promising complementary measures of specific neuronal correlates of consciousness.

More specifically, it was shown that auditory perceptual awareness is associated with an enhancement in the informational content of the neural signals from fronto-central brain areas and with an increase in the redundancy of the information in the temporal cortices. These results shows that the perceptual awareness of an auditory target can be characterized by variations in the macroscopic-scale neural signals and demonstrates the ability of manifold entropy measures and integrated information to discriminate conscious perceptual states on the basis of the informational content of neural signals.

## Figures and Tables

**Table 1:** Estimated marginal means for the post hoc multiple comparisons of the effects of marker density on detection performance index ($d^{\prime }$).

	Estimate	Std. Error	z Value	Pr(>|z|)
20–11	−0.39	0.12	−3.23	0.01
28–11	−0.34	0.12	−2.80	0.03
36–11	−0.11	0.12	−0.91	0.80
28–20	0.05	0.12	0.43	0.97
36–20	0.28	0.12	2.32	0.09
36–28	0.23	0.12	1.89	0.23

**Table 2:** Estimated marginal means of the post hoc paired comparisons between hit and miss trials for the two ERP components (ARN and P300) and their electrodes of interests. SE: standard error of the estimate; df: degrees of freedom (Kenward–Roger method). *p*-values were adjusted using Bonferroni method.

Electrode	Tone	Pairs	Estimate	SE	df	t-Ratio	*p*-Value
ARN							
	B2	H—M	0.697	0.338	81	2.05	0.170
	B1	H—M	−1.193	0.335	81	−3.56	0.002
C5	A1	H—M	0.154	0.323	81	0.47	1.000
	A2	H—M	0.341	0.346	81	0.98	1.000
	B2	H—M	0.308	0.300	95	1.02	1.000
	B1	H—M	−1.120	0.291	95	−3.84	<0.001
F6	A1	H—M	−0.341	0.288	95	−1.18	0.958
	A2	H—M	1.154	0.296	95	3.89	<0.001
	B2	H—M	0.517	0.335	90	1.54	0.505
	B1	H—M	−1.277	0.331	90	−3.85	<0.001
F7	A1	H—M	0.216	0.319	90	0.67	1.000
	A2	H—M	0.543	0.336	90	1.61	0.438
P300							
	B2	H—M	−0.156	0.145	101	−1.07	1.000
	B1	H—M	0.636	0.149	101	4.25	<0.001
FCz	A1	H—M	−0.034	0.143	101	−0.23	1.000
	A2	H—M	−0.445	0.143	101	−3.10	<0.01
	B2	H—M	−0.100	0.164	102	−0.61	1.000
	B1	H—M	0.780	0.166	102	4.69	<0.001
Cz	A1	H—M	−0.178	0.164	102	−1.08	1.000
	A2	H—M	−0.501	0.168	102	−2.97	<0.01
	B2	H—M	−0.546	0.173	99	−3.15	<0.01
	B1	H—M	0.999	0.177	99	5.63	<0.001
CPz	A1	H—M	−0.169	0.173	99	−0.97	1.000
	A2	H—M	−0.283	0.173	99	−1.63	0.42
	B2	H—M	−0.293	0.195	101	−1.50	0.546
	B1	H—M	1.003	0.198	101	5.06	<0.001
Pz	A1	H—M	−0.054	0.195	101	−0.27	1.00
	A2	H—M	−0.655	0.203	101	−3.21	<0.01

**Table 3:** Estimated marginal means of the post hoc paired comparisons between hit and miss trials in all the electrode clusters for the five entropy measures. SE: standard error of the estimate; df: degrees of freedom (Kenward–Roger method). *p*-values were adjusted using Bonferroni method.

Cluster	Measure	Pairs	Estimate	SE	df	t-Ratio	*p*-Value
Antero-Frontal	SpEn	H—M	−0.004	0.005	647	−0.727	1.000
	ApEn	H—M	−0.017	0.010	647	−1.817	0.627
	SaEn	H—M	−0.018	0.011	647	−1.706	0.796
	PeEn	H—M	−0.008	0.002	647	−3.388	0.006
	SvEn	H—M	−0.008	0.006	647	−1.265	1.000
Central	SpEn	H—M	−0.003	0.005	647	−0.588	1.000
	ApEn	H—M	−0.009	0.010	647	−0.981	1.000
	SaEn	H—M	−0.010	0.011	647	−0.903	1.000
	PeEn	H—M	−0.006	0.002	647	−2.526	0.106
	SvEn	H—M	−0.004	0.006	647	−0.627	1.000
Centro-Parietal	SpEn	H—M	−0.004	0.005	647	−0.817	1.000
	ApEn	H—M	−0.006	0.010	647	−0.679	1.000
	SaEn	H—M	−0.006	0.011	647	−0.571	1.000
	PeEn	H—M	−0.008	0.002	647	−3.351	0.007
	SvEn	H—M	−0.002	0.006	647	−0.253	1.000
Frontal	SpEn	H—M	−0.001	0.005	647	−0.177	1.000
	ApEn	H—M	−0.012	0.010	647	−1.290	1.000
	SaEn	H—M	−0.012	0.011	647	−1.169	1.000
	PeEn	H—M	−0.007	0.002	647	−2.834	0.042
	SvEn	H—M	−0.003	0.006	647	−0.588	1.000
Fronto-Central	SpEn	H—M	0.026	0.005	647	4.714	<0.001
	ApEn	H—M	0.083	0.010	647	8.709	<0.001
	SaEn	H—M	0.083	0.011	647	7.882	<0.001
	PeEn	H—M	0.060	0.002	647	24.726	<0.001
	SvEn	H—M	0.027	0.006	647	4.545	<0.001
Parietal	SpEn	H—M	−0.003	0.005	647	−0.527	1.000
	ApEn	H—M	−0.006	0.010	647	−0.582	1.000
	SaEn	H—M	−0.005	0.011	647	−0.501	1.000
	PeEn	H—M	−0.007	0.002	647	−2.937	0.030
	SvEn	H—M	−0.000	0.006	647	−0.045	1.000
Parieto-Occipital	SpEn	H—M	−0.003	0.005	647	−0.545	1.000
	ApEn	H—M	−0.011	0.010	647	−1.129	1.000
	SaEn	H—M	−0.011	0.011	647	−0.998	1.000
	PeEn	H—M	−0.009	0.002	647	−3.578	0.003
	SvEn	H—M	−0.003	0.006	647	−0.452	1.000
Sagittal	SpEn	H—M	−0.005	0.005	647	−0.881	1.000
	ApEn	H—M	−0.013	0.010	647	−1.400	1.000
	SaEn	H—M	−0.013	0.011	647	−1.237	1.000
	PeEn	H—M	−0.009	0.002	647	−3.536	0.003
	SvEn	H—M	−0.005	0.006	647	−0.911	1.000
Temporal	SpEn	H—M	−0.002	0.005	647	−0.452	1.000
	ApEn	H—M	−0.008	0.010	647	−0.831	1.000
	SaEn	H—M	−0.008	0.011	647	−0.797	1.000
	PeEn	H—M	−0.006	0.002	647	−2.577	0.091
	SvEn	H—M	−0.002	0.006	647	−0.405	1.000

## Data Availability

All data and codes supporting the results presented in this study will be available at https://osf.io/bqrac/ (accessed on 24 May 2023).

## Appendix A. Behavioral Results

**Table A1:** Summary of behavioral results for detection times and performance. For each subject (Subject Id.), the number of detected targets (Hit), undetected targets (Miss), false alarms (FA), and correct rejections (CR), as well as the correct detection rates (HIR), false alarm rates (FAR), detection performance indices $d^{\prime }$, the mean detection times (DT), and their standard deviation (DT σ) are indicated.

Subject Id.	Hit	Miss	FA	CR	HIR (%)	FAR (%)	d’	DT (ms)	DT σ (ms)
1	125	31	2	78	79	3	2.70	3016	1266
2	93	40	1	79	69	1	2.60	2928	1521
3	96	35	8	72	73	9	1.93	3277	1452
4	105	44	4	76	70	5	2.12	2874	1461
5	120	34	2	78	77	3	2.63	2631	1093
6	97	22	7	73	81	8	2.28	2891	1623
7	63	81	1	79	43	1	1.92	4006	1926
8	132	28	0	80	82	0	3.42	4046	1691
9	69	31	7	73	68	6	1.96	3003	1393
10	150	10	0	80	93	0	4.01	4467	2077
11	113	44	39	41	71	48	0.60	3609	1458
12	82	78	0	80	51	0	2.53	4629	1806
13	91	61	2	78	59	3	2.11	2693	1186
14	136	1	25	55	98	31	2.77	2843	1106
15	120	31	3	77	79	4	2.53	2857	1380
16	123	37	7	73	76	9	2.05	3391	1559
17	68	92	14	66	42	17	0.73	4540	2304
18	120	40	37	43	74	46	0.76	4849	1932
19	117	37	3	77	75	4	2.41	3318	1704
20	78	38	26	54	67	26	1.07	3115	1684
Total	2098	815	188	1412	—	—	—	—	—
Mean	105	41	9	71	71	11	2.15	3449	1581

## Appendix C. Entropy Measures

### Appendix C.1. Spectral Entropy: SpEn

The spectral entropy (SpEn), is a complexity marker characterizing the regularity of a temporal signal $x_{t}$ [119]. It is defined as the Shannon entropy of the spectral power density of the data and can be defined as follows:

1. Compute the spectrum $X(w_{i})$ of the signal.
2. Calculate the spectral power density of the signal via the square of its amplitude and normalize by the defined number of bins *N*.
  (A1) $$P\left(\omega_{i}\right)\;=\;\frac{1}{N}{\left|X\left(\omega_{i}\right)\right|}^{2}$$
3. Normalize the computed spectral power density so that it can be viewed as a probability mass function:
  (A2) $$p_{i}\;=\;\frac{P(\omega_{i})}{\sum_{i}P\left(\omega_{i}\right)}$$
4. SpEn can then be calculated using Shannon’s standard entropy formula.
  (A3) $$\mathrm{SpEn}\;=\;-\sum_{i=1}^{n}\;p_{i}\;\log p_{i}$$

### Appendix C.2. Approximate Entropy: ApEn

Approximate entropy (ApEn) is an information marker characterizing the regularity in the data fluctuations of a temporal signal $x_{t}$ [89,120]. It depends on several parameters; the main ones are the tolerance threshold *r*, under which a recurrence is found (also called filtering level), the length of the data vector considered *m* (also called integration dimension, see [121]), and time window duration *T*.

Approximate entropy (ApEn) is defined as follows: the time series is embedded in a phase space of vectors $X_{i}$ of delayed coordinates (called phases),

(A4) $$X_{i}\;=\;\left[x_{i},\;x_{i-1},\;\ldots \;,\;x_{i-m+1}\right]$$

where $x_{i}$ is the *i*-th sample of the studied time series. The correlation integral $C_{i}^{m}\left(r\right)$ indicates the probability that the integrated vector $X_{i}$ is similar to other vectors inside a threshold *r*:

(A5) $$C_{i}^{m}\left(r\right)\;=\;\frac{N_{i}^{r}}{N-m}\;i=1,\;\ldots \;,N-m+1$$

where *N* is the number of data samples and $N_{i}^{r}$ is the number of vectors whose distance to $X_{i}$ is less than *r*. The norm $L_{\infty }$ is chosen as the definition of the distance (i.e., the maximum distance between pairs of elements of the set of vectors).

Thus, the definition of the correlation integral $C_{i}^{m}\left(r\right)$ requires counting the number of recurrences $N_{i}^{r}$ of the trajectory towards points close to $X_{i}$ and dividing it by the number of pairs possible, thus estimating the percentage of neighboring points of $X_{i}$ (i.e., the probability that the trajectory has recurrences close to it). Finally, the approximate entropy ApEn is defined by the average degree of similarity $\phi^{m}\left(r\right)$ calculated on the basis of the correlation integral for two integration dimensions *m* and m+1:

(A6) $$\mathrm{ApEn}\;=\;\phi^{m}\left(r\right)-\phi^{m+1}\left(r\right)$$

with

(A7) $$\phi^{m}\left(r\right)\;=\;\frac{1}{\left(N-m+1\right)}\;\sum_{i=1}^{N-m+1}\;\log\;C_{i}^{m}\left(r\right)$$

The number of recurrences is higher in the lower dimension. Indeed, by increasing the dimension from *m* to m+1, an element is added to the vectors. This means that the recurrences in dimension m+1 are also recurrences in dimension *m*. It may happen that two vectors close in dimension *m* are not neighbors in dimension m+1, meaning that the last elements added to the two vectors are further apart than the tolerance threshold *r*.

The approximate entropy (ApEn) is therefore higher when the probability that the trajectories diverge is greater. Indeed, the logarithm in the definition of $\phi^{m}\left(r\right)$ is monotonic so that the entropy increases if the number of recurrences decreases when the dimension of integration is increased (from *m* to m + 1). The approximate entropy is thus largely influenced by the data length *N*, the tolerance threshold *r*, and the integration dimension *m*, and the following values are recommended: N=1000, *r* ranging from 0.1 to 0.25 % of the standard deviation of the signal, and m=2−3 [88,89].

### Appendix C.3. Sample Entropy: SaEn

Sample entropy (SaEn) is another information marker characterizing, like the approximate entropy, the data fluctuations of a temporal signal $x_{t}$ [122]. The approximate entropy (ApEn) has the bias of including potential self-similarity patterns in the data as well as being dependent on the size of the dataset. Sample entropy (SaEn) aims to compensate for these biases using a slightly different calculation procedure than the approximate entropy (ApEn). Sample entropy (SaEn) is defined as follows:

1. Let $\left[x_{1},\ldots ,\;x_{N}\right]$ be a time series of length *N*;
2. Let $X_{i}$ be the integrated series for 1≤i≤N−m+1, vectors of length *m*:
  (A8) $$X_{i}\;=\;\left[x_{i},\;x_{i+1},\;\ldots \;,\;x_{i+m-1}\right]$$
3. Let $n_{i}^{m}\left(r\right)$ be the number of vectors $x_{j}$ at a distance *r* from the vectors $x_{i}$, where j≠i and j=1,…,N−m+1 to exclude self-similar patterns;
4. Let $C_{i}^{m}\left(r\right)$, which is ${\left(N-m\right)}^{-1}$ times the number of $n_{i}^{m}\left(r\right)$, be defined as the probability that all $x_{j}$ is at a distance *r* from $x_{i}$;
5. Let $\phi^{m}\left(r\right)$ be the average degree of similarity that can be calculated as
  (A9) $$\phi^{m}\left(r\right)\;=\;\frac{\sum_{i=1}^{N-m+1}\log C_{i}^{m}\left(r\right)}{N-m+1}$$
6. Similarly, $\phi^{m+1}\left(r\right)$ can be computed for the embedding dimension of m+1:
  (A10) $$\mathrm{SaEn}\;=\;-\log\frac{\phi^{m+1}\left(r\right)}{\phi^{m}\left(r\right)}$$

where $\phi^{m}\left(r\right)$ represents the probability that two sequences correspond in dimension *m*, and $\phi^{m\;+\;1}\left(r\right)$ corresponds to the probability that two sequences correspond in dimension m+1.

In this way, the sample entropy (SaEn) does not include self-similar patterns and does not depend on the data size. Parameter values similar to approximate entropy (ApE)) are recommended in the literature for sample entropy (SaEn) [90,91,92].

### Appendix C.4. Permutation Entropy: PeEn

The permutation entropy (PeEn) is an information marker capturing the order relations between the values of a temporal signal $x_{t}$ associated with a dynamic system [123]. The time signal is transformed into a sequence of discrete symbols, and the entropy of the signal is quantified from the probability densities of these symbols. The transformation is performed by extracting sub-vectors from the signal, each comprising *m* measurements separated by a fixed time delay τ. Similar to ApEn and SaEn, the permutation entropy (PeEn) is based on three parameters: the embedding dimension *m*, the embedding delay τ, and the length of the signal *N*. The permutation entropy (PeEn) can be calculated as follows:

1. Given an input time series $\left[x_{0},\;x_{1},\;\ldots \;,\;x_{N-1}\right]$, and an embedding dimension m>1;
2. For each subsequence extracted at time *t*, $\left[x_{t-(m-1)},\;x_{t-(m-2)},\;\ldots \;,\;x_{t-1},\;x_{t}\right]$, a rank model π relative to *t* is obtained in the form $\pi =[r_{0},\;r_{1},\;\ldots \;,\;r_{m-1}]$;
3. This rank pattern is defined by an order pattern $x_{t-r_{m-1}}\;\leq \;x_{t-r_{m-2}}\;\leq \;\ldots \;\leq \;x_{t-r_{1}}\leq \;x_{t-r_{0}}$;
4. For all possible m! permutations, each probability p(π) is estimated as the relative frequency of each different pattern π found;
5. Once all these probabilities have been obtained, the final value of the permutation entropy (PeEn) is given by
  (A11) $$\mathrm{PeEn}\;=\;-\sum_{j=0}^{m!-1}\;p\left(\pi_{j}\right)\;\log\;p\left(\pi_{j}\right)$$

The parameter values recommended in the literature for the permutation entropy (PeEn) are 3≤m≤7, τ=1 and N>>m! [123,124].

### Appendix C.5. Singular-Value Decomposition Entropy: SvEn

The singular-value decomposition entropy (SvEn) is an information marker characterizing the data dimension of a temporal signal $x_{t}$ [125,126] and represents a tool that can complement the existing nonlinear analysis methods in order to test the complexity of the time series [127].

It indicates the number of eigenvectors necessary for an adequate explanation of the data associated with the signal. Globally, the singular-value decomposition entropy (SvEn) is calculated from the distribution of the singular values of the matrix *M*, including all the vectors constructed according to a delay procedure. First, we construct a vector of delays $y_{i}$ on the basis of a time signal $\left[x_{1},x_{2},\ldots ,x_{n}\right]$ such that

(A12) $$y_{i}\;=\;\left[x_{i},\;x_{i+\tau },\;\ldots \;,\;x_{i+(m-1)*\tau }\right]$$

where τ corresponds to the delay considered and *m* to the embedding dimension.

We then build an embedding matrix *Y* such that

(A13) $$Y\;=\;{\left[y_{i},\;y_{2},\;\ldots \;,\;x_{N-(m-1)*\tau }\right]}^{T}$$

A singular-value decomposition is then performed on matrix *Y* in order to produce *M* values $\sigma_{1},\sigma_{2},\ldots ,\sigma_{M}$, which represent a singular spectrum of values.

The singular-value decomposition entropy (SvEn) can then be defined as

(A14) $$\mathrm{SvEn}\;=\;-\sum_{i=1}^{M}\;\sigma_{i}\;\log\;\sigma_{i}$$

## Appendix D. Integrated Information Measures

### Appendix D.1. Decoding-Based Integrated Information *Φ**

Integrated information measures aim to characterize the difference in information between an effective system and its interactions and a totally independent system. This difference aims to fully quantify the integration of information in an organized system, such as the brain. Each measure approaches the problem according to a particular theoretical point of view, giving it its specificity.

First, integrated information based on decoding Φ* approaches the problem in terms of the transmitter/receiver by considering their situation: the first one corresponds to that where the receiver decodes the system information from the probability distribution corresponding to that observed, and compares it to the second situation, where the receiver decodes the message on the basis of a probability distribution resulting from a system whose parts are completely independent.

That can be briefly formalized as

(A15) $$\Phi^{*}\;=\;IM\left(X_{t};X_{t-\tau }\right)-IM^{*}\left(X_{t};X_{t-\tau }\right)$$

where τ is the desired time delay to characterize the integration of information in the system, $IM(X_{t};X_{t-\tau })$ is the mutual information between the present states and the past states delayed according to τ, and $IM^{*}$ is the decoding information from the system to the independent parties.

The complete formalism for the computation of Φ* is presented in [67].

### Appendix D.2. Geometric Integrated Information *Φ*^G^

Geometric integrated information $\Phi^{G}$ uses information geometry, which is an application of differential geometry to the relationships and structures of probability distributions. In this formalism, the Kullback–Leibler divergence is a natural measure of the distance between two probability distributions. The geometric integrated information $\Phi^{G}$ is based on this fact and aims to measure the distance between the probability distribution of the system compared to that of a totally disconnected system.

This can be briefly summarized as

(A16) $$\Phi^{G}\;=\;\min_{q}\;D_{KL}\left[p\left(X_{t},X_{t-\tau }\right)\;\left|\right|\;q\left(X_{t},X_{t-\tau }\right)\right]$$

where $D_{KL}\left[p||q\right]$ represents the Kullback–Leibler divergence between the two joint probability distributions of the present and past states of the system between the connected *p* and disconnected *q* models.

The complete formalism for the computation of $\Phi^{G}$ is presented in [69].

### Appendix D.3. Stochastic Integrated Information *Φ*^H^

The stochastic integrated information $\Phi^{H}$ approaches the problem from the probabilities of transition between one state and another in the case of the integrated system compared to a system whose states evolve completely independently.

That can be understood as

(A17) $$\Phi^{H}\;=\;\sum_{i}H\left(M_{t-\tau }^{i}|M_{t}^{i}\right)-H\left(X_{t-\tau }|X_{t}\right)$$

where $H(X_{t-\tau }|X_{t})$ measures the amount of information “lost” irreversibly, and $M^{i}$ are the states of the system.

The complete formalism for the computation of $\Phi^{H}$ is presented in [70,96].

### Appendix D.4. Redundancy-Based Integrated Information or “Multi-Information” *Φ*^MI^

The multi-information $\Phi^{MI}$, also called “multivariate mutual information“ [128,129], represents a multivariate generalization of Shannon’s mutual information [130,131].

It quantifies the integration of information by measuring the amount of information shared by the variables of the connected system compared to that shared when all interactions are removed, that is, when the *M* parts of the system are considered independent [70]:

(A18) $$\Phi^{MI}\;=\;\sum_{i}\;H\left(M_{t-\tau }^{i},M_{t}^{i}\right)-H\left(X_{t-\tau },X_{t}\right)$$

where $H(X_{t-\tau },X_{t})$ is the joint entropy.

The complete formalism for the computation of $\Phi^{MI}$ is presented in [70].
